# Phenotypic and genotypic characterization of *Neisseria gonorrhoeae* isolates from Ethiopia, 2021 to 2023

**DOI:** 10.1093/jacamr/dlag126

**Published:** 2026-07-23

**Authors:** Mulatu Melese Derebe, QinQin Yu, Abaineh Munshea, Gizachew Yismaw Wubetu, Surafel Fentaw, Tesfa Addis Kefale, Rebecca M McSweeney, Nadia Debech, Vegard Eldholm, Tatiana Ponton Tomaselli, Afework Kassu, Adane Mihret, Yemane Berhane, Anne C C Lee, Yonatan H Grad, Bente Børud

**Affiliations:** Health Biotechnology Division, Institute of Biotechnology, Bahir Dar University, Bahir Dar, Ethiopia; Amhara Public Health Institute, Bahir Dar, Ethiopia; Department of Immunology and Infectious Diseases, Harvard T.H. Chan School of Public Health, Boston, USA; Health Biotechnology Division, Institute of Biotechnology, Bahir Dar University, Bahir Dar, Ethiopia; Amhara Public Health Institute, Bahir Dar, Ethiopia; National Clinical Bacteriology and Mycology Reference Laboratory, Ethiopian Public Health Institute, Addis Ababa, Ethiopia; National Clinical Bacteriology and Mycology Reference Laboratory, Ethiopian Public Health Institute, Addis Ababa, Ethiopia; Department of Immunology and Infectious Diseases, Harvard T.H. Chan School of Public Health, Boston, USA; Department of Bacteriology, Norwegian Institute of Public Health, Oslo, Norway; Department of Bacteriology, Norwegian Institute of Public Health, Oslo, Norway; Department of Bacteriology, Norwegian Institute of Public Health, Oslo, Norway; Armauer Hansen Research Institute, Addis Ababa, Ethiopia; Armauer Hansen Research Institute, Addis Ababa, Ethiopia; Addis Continental Institute of Public Health, Addis Ababa, Ethiopia; Department of Pediatrics, Global Alliance for Infant and Maternal Health Research, Warren Alpert Medical School, Brown University, Providence, RI, USA; Department of Immunology and Infectious Diseases, Harvard T.H. Chan School of Public Health, Boston, USA; Department of Bacteriology, Norwegian Institute of Public Health, Oslo, Norway

## Abstract

**Objectives:**

Antimicrobial resistance (AMR) in *Neisseria gonorrhoeae* is increasing globally, but some regions remain understudied. This study aimed to investigate the AMR phenotypes and genotypes of *N. gonorrhoeae* isolates from Bahir Dar, Ethiopia, and their phylogenetic relationship to global isolates.

**Methods:**

Endocervical and urethral swabs were collected from symptomatic patients at sexually transmitted infection (STI) clinics from September 2021 to April 2023. The identified *N. gonorrhoeae* isolates (*n* = 70) were subjected to AMR phenotyping and WGS. We generated a recombination-corrected phylogeny with 724 global representative isolates and used phylodynamics to estimate the introduction times of isolates into Bahir Dar.

**Results:**

All isolates were resistant to ciprofloxacin and tetracycline but susceptible to ceftriaxone, cefixime, azithromycin, and spectinomycin (100% susceptibility); 67 (95.7%) were resistant to penicillin. WGS identified 14 MLSTs, with ST-1587 (54.3%) the dominant sequence type. AMR determinants correlated with phenotypic findings for all antimicrobials. The phylogenetic analysis suggested at least nine introductions of *N. gonorrhoeae* into Bahir Dar. The ST-1587 isolates were introduced recently, showing close genetic ties to global isolates sampled in 2018–2019 from Asia, Europe, North America, and Oceania. ST-7827 displayed greater genetic divergence from global isolates.

**Conclusions:**

This study presents WGS of *N. gonorrhoeae* isolates from Ethiopia, uncovering diverse sequence types and multidrug resistance. These findings underscore the need for routine genomic surveillance, improved antimicrobial stewardship, and targeted public health interventions to combat the spread of AMR and improve STI management in Ethiopia.

## Introduction

*Neisseria gonorrhoeae* infection is a global health concern due to the disease burden and increasing antimicrobial resistance (AMR). In 2020, the WHO estimated that 82.4 million new cases of gonorrhoea occur each year,^1^ with the highest prevalence reported in the WHO African and Western Pacific regions.^2^ Surveillance is crucial for identifying emerging AMR patterns and guiding effective STI treatment strategies. Surveillance of *N. gonorrhoeae* infections remains limited in Africa, with only eight of the 47 African countries reporting to the WHO Gonococcal AMR Surveillance Programme during 2019–2022.^3^

Prompt and effective antimicrobial treatment is crucial to prevent serious complications. In Ethiopia, STI management usually follows a syndromic approach. For symptomatic urethral and vaginal discharge, Ethiopia's national guidelines recommend ceftriaxone (250 mg intramuscularly) combined with azithromycin (1 g orally) as first-line treatments to address mixed infections of *N. gonorrhoeae* and *Chlamydia trachomatis,* given the limited availability of laboratory confirmation.^4^ However, the Ethiopian guidelines, which have remained unchanged since 2015, do not reflect the latest WHO recommendation of a single 1 g intramuscular dose of ceftriaxone.^5^

A study of gonorrhoea in five Ethiopian cities from 2021 to 2022 provided the first national data on phenotypic antimicrobial susceptibility, along with epidemiological data.^6^ This study reported that all 299 isolates were susceptible to ceftriaxone, cefixime, azithromycin, and spectinomycin, and a high proportion were resistant to ciprofloxacin, tetracycline, and benzylpenicillin. However, genome sequencing was not performed on these isolates.

WGS of *N. gonorrhoeae* isolates provides valuable insights into the circulating gonococcal population and the genetic determinants of AMR.^7^ WGS-based data also enable phylogenetic analysis of *N. gonorrhoeae* to understand the genetic diversity, population structure, and global spread of gonococcal lineages with AMR.^8^ While several studies have reported *N. gonorrhoeae* genome sequences from various regions within Africa,^9–12^ contributing to global gonococcal genomic research, there remains a notable underrepresentation of genomic data specifically from Ethiopia. To address this gap, we conducted whole-genome sequencing of 70 *N. gonorrhoeae* isolates collected from Bahir Dar, Ethiopia, between September 2021 and April 2023.

## Materials and methods

### Ethics

This study was conducted in accordance with the national and institutional research standards. Ethical approval was obtained from the Bahir Dar University College of Science Ethics Committee (Ref No: PRCSVD/168/213). Additional approval and an official support letter were provided by the Amhara Regional State Public Health Institute. Written informed consent was obtained from all participants prior to data collection. Patient demographic data, including age, sex, anatomical site of sample collection, and travel history in the past two weeks, were also recorded.

### Study participants

**Figure 1.**
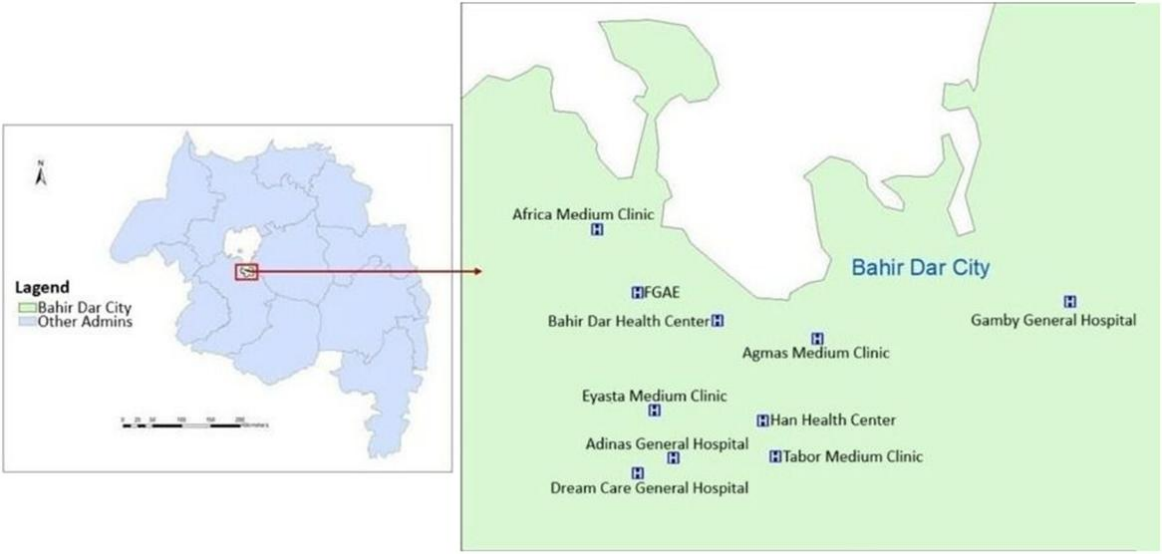
The selected study sites for sample collection included 10 STI clinics in Bahir Dar, Amhara Region, Ethiopia (2021–2023). EGAE; Family Guidance Association of Ethiopia.

Participants were recruited from STIs and outpatient clinics in and around Bahir Dar, the capital of Amhara region, Ethiopia. The participating clinics included the Family Guidance Association of Ethiopia (FGAE), Agmas Medium Clinic, Africa Medium Clinic, Adinas General Hospital, Tabor Medium Clinic, Dream Care General Hospital, Bahir Dar Health Centre, Han Health Centre, GAMBY General and Teaching Hospital, and Eyasta Specialty Medical Centre (Figure 1). A standardized set of clinical indicators (urethral or vaginal discharge, painful urination, and genital discomfort) was employed to identify patients presenting with symptoms potentially indicative of gonorrhoea infection, in accordance with the Ethiopian national syndromic management protocols.^4^

### Isolate collection and processing

Samples were collected before initiating any antibiotic treatment. Two swabs were collected from each of 431 patients and transported to the Amhara Public Health Institute (APHI) Microbiology Reference Laboratory for analysis. One swab was tested with the GeneXpert^®^ CT/NG assay (Cepheid, Sunnyvale, CA), while the other was inoculated onto Modified Thayer-Martin agar plates and incubated at 37°C in 5% CO_2_ for 24–48 h. In 169 of the samples, *N. gonorrhoeae* was confirmed by the Xpert^®^ CT/NG assay, and of these, 90 were successfully cultured. *N. gonorrhoeae* isolates were confirmed microscopically as Gram-negative diplococci. Isolates were sub-cultured on chocolate agar, verified using catalase and oxidase tests, and stored in Tryptone Soya Broth (TSB) with glycerol at −80°C (25% glycerol was made in TSB as working solution). These isolates were shipped on dry ice to the Norwegian Institute of Public Health (NIPH) for whole-genome sequencing.

At the NIPH Microbiology Reference Laboratory, isolates were cultured on Neisseria selective agar (Thermo ScientificTM) at 37°C in 5% CO2 for 18–24 h. Colonies exhibiting *N. gonorrhoeae* morphology were subcultured on chocolate agar (GC II Agar with IsoVitaleX, Becton Dickinson Norway AS) under the same conditions. Pure isolates (*n* = 70) were confirmed as *N. gonorrhoeae* using the MALDI-TOF ^®^Microflex ^®^LRF System (Brüker), with identification performed with the software packages ‘Flexcontrol’ and ‘MBT Compass’ (MALDI Biotyper Compass) using the BDAL-Brüker standard library for reference.

### Antimicrobial susceptibility testing

Beta-lactamase production was determined using Cefinase patches with chromogenic cephalosporin nitrocefin (BBL Cefinase, BD). AST was performed using Etests for Penicillin G, Cefixime, Ceftriaxone, Tetracycline, Ciprofloxacin, Azithromycin, and Spectinomycin, following manufacturer instructions (bioMérieux, Marcy-l'Étoile, France). Minimum inhibitory concentrations (MICs) were determined by measuring growth inhibition zones around the Etest strips, with MIC values read at every twofold dilution. Results were interpreted as susceptible, intermediate, or resistant (S, I, R) based on EUCAST clinical breakpoints v 14.0.

### Whole-genome sequencing, assembly, and analysis

Genomic DNA was extracted from pure bacterial culture using the MagNA Pure 96 system (Roche Life Science) and quantified with a Fluoroskan microplate fluorometer (Thermo Scientific). Sequencing libraries were prepared using the xGen DNA Library Prep EZ UNI kit with full-length unique dual-indexed adapters (Integrated DNA Technologies, IDT). The quality of prepared DNA libraries was assessed by measuring DNA concentration and fragment size using Fluoroskan and the TapeStation 4200 system (Agilent), respectively. Libraries were sequenced on an Illumina NextSeq 550 platform using the NextSeq 500/550 Mid Output Kit v2.5 (300 cycles).

Sequencing data were trimmed using Trimmomatic (v0.390)^13^ and assembled using the *de novo* assembler SPAdes (v3.14.1).^14^ Low quality contigs (length < 500 bp and coverage < 2 kmer) were removed using an in-house filtering script. Quality control of the sequencing data was performed using MultiQC (v1.9), while species verification of the assemblies was conducted using Kraken (v1.1.1). Quality assessment of raw sequencing data and assembled contigs was performed using MultiQC (v1.9) software,^15^ while the quality of the assembled FASTA contigs was verified using Kraken (v1.1.1).^16^

Isolates were characterized by sequence typing using the PubMLST *Neisseria* MLST scheme^17^ and the NG-STAR allelic and profile definitions extracted from the NG-STAR database (https://ngstar.canada.ca/).^18^ The cgMLST clustering and phylogenetic tree construction were performed using Pathogenwatch (https://pathogen.watch/)^19^ and visualized with Phandango.^20^ Pathogenwatch was also used to predict AMR, which was obtained using PAARSNa AMR-Library 485 version 0.0.17. Genome data generated in this study were deposited in the PubMLST.org database (http://pubmlst.org/neisseria/), identification numbers and PubMLST isolate names are available in Table S1 (available as Supplementary data at *JAC-AMR* Online).

### Global dataset and genome assembly

Publicly available WGS data (*n* = 24 262) from clinical *N. gonorrhoeae* isolates were retrieved from the European Nucleotide Archive (ENA), and the date and geographic location of isolation were extracted from the associated publications. Reference-based genome assembly, *de novo* genome assembly, and quality control filtering followed previously described methods.^21,22^ A total of 21 583 genomes meeting quality control filtering were included in the study (Table S2 and https://github.com/gradlab/ethiopia_gc/blob/main/data/isolates_summary_and_qc/global_isolates_metadata_met_qc.csv).

### Selection of representative global genomes

The global dataset of *N. gonorrhoeae* genomes was clustered using PopPUNK v 2.6.0.^23^ The isolate in each PopPUNK cluster with the fewest contigs in its *de novo* assembly was chosen as the representative isolate from that cluster, resulting in 724 representative genomes. Accessions and quality control statistics for the representative genomes are available at github.com/gradlab/ethiopia_gc/tree/main/data/isolates_summary_and_qc/representative_isolates_accession_and_qc.csv.

### Phylogenies of representative global genomes, ST-1587 genomes, and ST-7827 genomes

The MLST and NG-STAR profiles of all global genomes were determined using pyngoST v 1.1.2^24^ applied to the *de novo* assemblies. The reference-mapped pseudogenomes were used to build recombination-masked phylogenies with Gubbins v 3.3.4,^25^ applying the GTR substitution model and RAxML Next Generation tree-building algorithm.^26^ The resulting phylogenetic trees were midpoint rooted and visualized using iTOL v 6.9.1.^27^

### Estimation of the time of divergence

A time-scaled phylogeny was reconstructed in BEAST2 v 2.7.6^28^ using the alignment of recombination-filtered polymorphic sites from Gubbins and corresponding sampling dates. This analysis employed the GTR substitution model, gamma-distributed site rate heterogeneity with four categories, and a relaxed molecular clock model to infer the divergence time.

### Assessment of gene content differences

Gene content differences across genomes were assessed through pangenome analysis of the global representative isolates and the study isolates using Panaroo v 1.5.0,^29^ using the strict clean mode option. The presence of the Gonococcal Genetic Island (GGI) was determined using BLAST v 2.14.1 as described in Youngblom *et al*.^30^ Nucleotide sequences of the 61 GGI genes annotated in *N. gonorrhoeae* strain MS11 (ranging from *traD* to *parA*, accessible at https://www.ncbi.nlm.nih.gov/nuccore/AY803022.1) were used as reference sequences to identify GGI genes. Only alignments from the BLAST results covering at least 50% of the gene’s full length were included in the analysis. Data postprocessing was performed using Python v3.11.4.

## Results

### *Neisseria gonorrhoeae* sampling

Samples were collected from 431 symptomatic patients attending 10 STI clinics in Bahir Dar, Northwest Ethiopia (Figure 1), between September 2021 and April 2023. A total of 169 *N. gonorrhoeae* positive cases were detected using GeneXpert from 431 symptomatic patients (39%) who presented with urethral or vaginal discharge, painful urination, or genital discomfort. At APHI, 90 of 169 (53%) *N. gonorrhoeae* isolates were identified by culture from endocervical or urethral swabs collected from symptomatic patients, confirmed as Gram-negative diplococci, and verified with catalase and oxidase tests. Of these, 70 isolates (78%) were successfully cultured, sequenced, and analysed at NIPH. The patients’ ages ranged from 17 to 35 years, with 42 isolates (60%) obtained from males and 28 (40%) from females. Twenty-three participants (32.9%) reported having four or more sexual partners within the previous 6 months, and 13 (18.6%) reported recent local travel (Table S3).

### Antibiotic susceptibility in Bahir Dar, Ethiopia

Susceptibility to seven antimicrobials was evaluated (Table 1). All isolates were susceptible to cefixime, ceftriaxone, azithromycin, and spectinomycin and resistant to tetracycline and ciprofloxacin. Sixty-seven isolates (95.7%) were resistant to penicillin, with an MIC range of 2 to >32 mg/L and were β-lactamase producers; the remaining three isolates exhibited intermediate resistance to penicillin.

**Table 1.** Antimicrobial susceptibility profiles of *N. gonorrhoeae* isolates, Bahir Dar, Ethiopia (2021–2023)

Antimicrobials	Isolates (%) MIC range for the isolates (mg/L)		
	Sensitive	Intermediate	Resistant
Penicillin G (PCN)	0 (0%)	3 (4.3%) 0.125–0.25	67 (95.7%) 2 - > 32
Ciprofloxacin (CIP)	0 (0%)	0 (0%)	70 (100%) 0.5–8
Cefixime (CFM)	70 (100%) ≤0.016		0 (0%)
Ceftriaxone (CRO)	70 (100%) <0.016		0 (0%)
Tetracycline (TET)	0 (0%)		70 (100%) 8–32
Spectinomycin (SPX)	70 (100%) 8–32		0 (0%)
Azithromycin (AZM)	70 (100%)^a^ 0.016–0.25		

^a^For testing purposes to detect acquired resistance mechanisms, the ECOFF is 1 mg/L.

### Molecular epidemiology and AMR determinants

**Figure 2.**
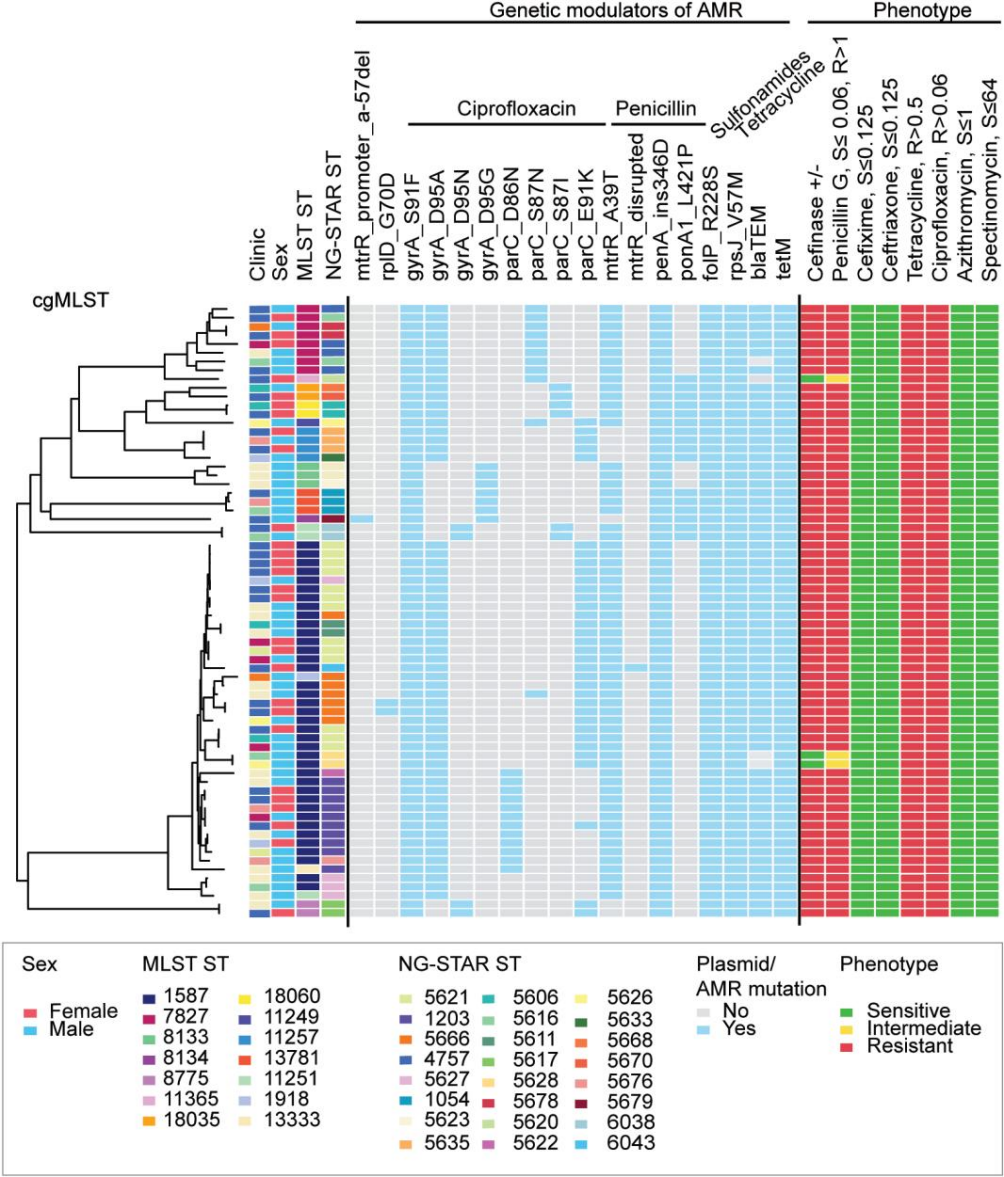
Core genome MLST, AMR genotypes, and phenotypes of *N. gonorrhoeae* isolates. An overview of the phylogenetic relationship and AMR phenotypes and genotypes for this strain collection. All data points used in this figure are available in Table S1. The isolates were identified by their PubMLST isolate names within the cgMLST tree. The colour codes for sex, MLST ST, AMR genotype and phenotype, and NG-STAR mutations are shown in the figure legend. Refer to Table S1 for precise MIC values and clinic names. The frequency of resistant isolates and various mutations is presented in Table 2.

As of August 2025, > 28 000 *N. gonorrhoeae* genomes are available on PubMLST, but African isolates are undersampled, constituting only 3.8% of entries (PUBMLST.org/neisseria). In this study, all isolates were characterized using MLST from PubMLST,^31^ NG-STAR,^18^ and Pathogenwatch (Figure 2). A total of 14 sequence types (STs) were identified. The most frequent were ST-1587 (54.3%), ST-7827 (11.4%), and ST-11257 (5.7%), while the remaining STs comprised only 1–3 isolates each. ST-1587, consisting of 38 isolates, was the only sequence type with sufficient numbers for further analysis. These isolates were predominantly collected from two clinics, 26 (37.1%) from FGAE and 16 (22.8%) from Agmas clinic; the rest were distributed across eight clinics (Table S4). The gender distribution within ST-1587 was 21 male and 17 female cases (Table S5).

A total of 24 NG-STAR sequence types were identified, with NG-STAR ST-5621 (18.6%), ST-1203 (14.3%), and ST-5666 (10%) the most common. The remaining NG-STAR STs accounted for 1–4 isolates each (Table S6, Figure 2). NG-STAR sequence types were evenly distributed across clinics (Table S7).

The genome-based AMR predictions using Pathogenwatch aligned well with the phenotypic resistance profiles for all six antimicrobials tested (Figure 2). Mutations associated with resistance to penicillin, ciprofloxacin, tetracycline, and sulphonamides were commonly observed, and alleles of the resistance-related genes are shown in Table 2. All isolates were tetracycline-resistant, carrying both the *tetM* plasmid and the *rpsJ* V57M mutation. Sulphonamide resistance, linked to the *folP* R228S mutation, was found in all isolates. Mutations in the *mtrR* gene, which result in upregulation of the MtrCDE efflux pump, were frequent, with 90% of isolates carrying the A39T mutation, while only one isolate had a promoter deletion, and another had a gene disruption. Ciprofloxacin resistance was characterized by the *gyrA* S91F mutation and mutations at codon D95 (mostly D95A), alongside diverse *parC* mutations, with E91K the most common. Most isolates (95.7%) were resistant to Penicillin-G, and 94.3% harboured the *blaTEM* plasmid. All isolates had *penA* ins346D mutation, and 22.9% also had the *ponA* L421P mutation associated with penicillin resistance (Figure 2, Table 2).

**Table 2.** Resistance-related genes of *N. gonorrhoeae* isolates collected from Bahir Dar, Ethiopia (2021–2023)

Gene	Locus (PubMLST)	Alleles in the Bahir Dar isolates collection	AMR-associated amino acid substitution (% of isolates)
*penA*	NEIS1753	2.001	
		2.002	
		2.008	
		9.001	Ins346D (100%)
		19.001	
		19.021	
		277.001	
*mtrR*	^Pro^NEIS1635	1 (−35A Del)	
		5 (wt)	−35A Del (1.4%)
		10 (A39T)	
	NEIS1635	54 (A39T)	
		89 (A39T)	
		649 (A39T)	A39T (90%)
		166 (A39T)	
		605 (A39T)	
*porB1b*	NEIS2020	3 (A121S)	
		13 (porB1a)	
		14 (porB1a)	
		37 (G120N, A121G)	—
		100 (wt)	
*ponA*	NEIS 0414	1 (L421P)	
		100 (wt)	L421P (22.9%)
*gyrA*	NEIS1320	1 (S91F, D95G)	S91F (100%)
		2 (S91F, D95N)	D95A (84.3%)
		7 (S91F, D95A)	D95N (5.7%)
			D95G (10%)
*parC*	NEIS1525	4 (D86N)	D86N (17.1%)
		22 (wt)	S87N (14.3%)
		26 (S87N, E91K)	S87I (8.6%)
		40 (E91K)	E91K (47.1%)
		49 (wt)	
		103 (S87N)	
		131 (S87I)	
		194 (D86N)	
		195 (S87N)	
23S rRNA		6 (wt)	-
		100 (wt)	

### Phylogenetic analysis with global representative isolates

To better understand how these isolates are related to the globally circulating *N. gonorrhoeae*, we compiled a set of 724 genomes, reflecting the global diversity of publicly available *N. gonorrhoeae* genomes. First, we evaluated gene content across the study and international isolates and found that all genes in the study isolates were also present in the international isolates, except one unique accessory gene in Eth03-2021 that encodes for a hypothetical protein. The GGI is present in 6/70 (9%) of isolates, maintaining 98–100% of GGI genes per isolate.

**Figure 3.**
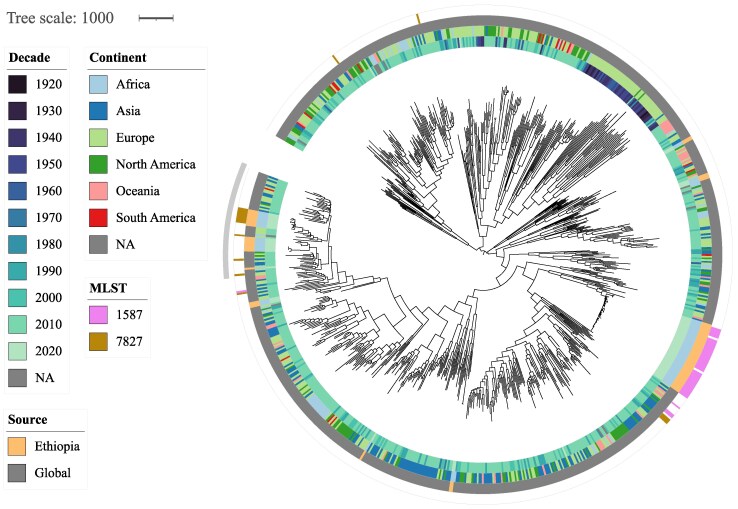
The Bahir Dar isolates in a recombination-corrected phylogeny with 724 global *N. gonorrhoeae* isolates. The tree scale is given in the number of recombination-corrected mutations. The grey shaded arc on the outer ring indicates the isolates that were used to estimate the time of divergence of Ethiopian ST-7827 isolates from global isolates.

We generated a recombination-corrected phylogenetic tree of the isolates from this study with representative global isolates (Figure 3). Based on the sampled isolates, we inferred at least 9 introductions of *N. gonorrhoeae* into Bahir Dar. The time of introduction of the isolates varied. For example, ST-1587 isolates were introduced relatively recently, as they are closely related to isolates sampled in 2018–2019 from Asia, Europe, North America, and Oceania (Figure S1). In contrast, the ST-7827 isolates are more divergent from global ST-7827 isolates (Figure S2); however, ST-7287 isolates from Bahir Dar are more closely related to isolates in other STs than to ST-7827 from outside of Ethiopia, as can be seen in the lengths of the branches in the phylogeny with the global isolates (Figure 3). To estimate the time of divergence, the most closely related genomes (*n* = 48) to the Ethiopian ST-7827 isolates with known sampling dates were identified from both the representative global and Ethiopian isolates (see grey shaded arc in Figure 3). We estimated the time of divergence of the ST-7827 Bahir Dar isolates from the closest non-ST-7827 Ethiopian isolate to be around 2012 [95% highest posterior density (HPD) 2004–2018] and from the closest global isolates to be around 2002 (95% HPD 1991–2011) (Figure S3).

## Discussion

This study investigated the phenotypes and genetic determinants of AMR exhibited by gonococcal isolates in Bahir Dar, Ethiopia, between 2021 and 2023. *N. gonorrhoeae* culture is essential for antimicrobial susceptibility testing and molecular surveillance but poses challenges due to its fastidious growth requirements and similarities to other *Neisseria* species. Consequently, nucleic acid amplification tests (NAATs) offer higher specificity and sensitivity. In this study, 53% of *N. gonorrhoeae* NAAT-positive cases were successfully cultured in Ethiopia, with 78% of these recovered and included in the analysis. All 70 isolates exhibited phenotypic resistance to ciprofloxacin and tetracycline, with 95.7% also resistant to penicillin, while susceptibility was observed for ceftriaxone, cefixime, azithromycin, and spectinomycin for all isolates. Genotypic AMR determinants aligned with phenotypes.

Our findings align with studies conducted in other parts of Ethiopia,^6,32–34^ as well as reports from the WHO and the Gonococcal Antimicrobial Surveillance Programme.^3,35^ Similar resistance profiles have been reported in neighbouring Kenya^36^ and Uganda,^37^  and West African countries such as Côte d’Ivoire,^38^ Guinea-Bissau,^39^ Benin,^40^ and Ghana.^11^ Sustained AMR surveillance is key to detecting any emerging resistance and ensuring that treatment guidelines remain effective in both local and global settings.^41^

The recommended initial treatment for gonorrhea in Ethiopia consists of ceftriaxone and azithromycin, which has remained unchanged since 2015,^4^ and does not align with the latest WHO recommendation for higher dose ceftriaxone monotherapy.^5^ Encouragingly, all isolates demonstrate low MIC values for ceftriaxone and cefixime (≤0.016 mg/L), as well as for azithromycin (≤0.25 mg/L). Nevertheless, there are increasing reports of resistance or reduced susceptibility to these antimicrobials on a global scale.^3^ The limited capacity for routine culture and antimicrobial susceptibility testing in Ethiopia, along with the ongoing use of syndromic management approaches, may further drive the selection and transmission of resistant *N. gonorrhoeae* strains. The country has a One Health strategic plan aimed at preventing and containing AMR.^42^ However, the implementation of these initiatives remains inconsistent across healthcare facilities. Challenges such as resource limitations, diagnostic constraints, interruptions in programme continuity, and gaps in the national AMR surveillance systems continue to hinder the full effectiveness of antimicrobial stewardship interventions.

MLST and NG-STAR analysis revealed substantial genetic diversity among the isolates, identifying 14 STs and 24 NG-STAR sequence types. ST-7827 and ST-11257 were the second most prevalent sequence types identified in this study. All isolates carried the *tetM-*encoding plasmid and the chromosomal *rpsJ* V57M mutation and had tetracycline MICs ≥8 mg/L, indicating doxycycline post-exposure prophylaxis (doxy-PEP) is expected to be ineffective for gonorrhoea in this population.^43^

Mutations in the *mtrR* gene were also observed, specifically the A39T substitution found in 90% of isolates and a −35A deletion in the promoter region identified in one isolate. These mutations are known to upregulate the MtrCDE efflux pump, contributing to multidrug resistance, including resistance to β-lactams.^44^ Despite azithromycin being part of the recommended treatment, no azithromycin resistance was observed (Table 2 and Figure 2). These findings are consistent with recent WHO surveillance data from different African countries, indicating that extended-spectrum cephalosporins (ESCs) largely remain effective against gonococcal strains.^3,12^ Nevertheless, the global rise of ESC-resistant clones highlights the need for ongoing molecular surveillance.^35^

While overall gene content was similar between Bahir Dar and global isolates, there were some differences in specific genes. All genes in the isolates from Bahir Dar were present in the 724 globally representative genomes, except for one unique accessory gene identified in Eth03-2021. The GGI, a horizontally acquired element implicated in increased virulence and genetic competence of *N. gonorrhoeae,*^45^ was detected in only 9% of the Bahir Dar isolates, substantially lower than reported in other studies, such as 61% in New York, USA^30^ and 64% in the Netherlands.^46^ The low prevalence of GGI observed in this study could be influenced by various factors, including the types of isolates sampled, differences in circulating strains in this region compared with other locations, and possible variation in selection pressures. Further studies would be needed to determine the underlying reasons.

The phylogenetic analysis revealed at least nine inferred independent introductions of *N. gonorrhoeae* into Bahir Dar and in some cases clonal expansion within the region (Figure 3). The dominant sequence type in this study, ST-1587, was closely related to global isolates from Asia, Europe, North America, and Oceania, sampled between 2018 and 2019 (Figure S1), and shows recent introduction into Ethiopia. Ethiopian ST-7827 isolates formed a separate, deeply branching clade when analysed together with a global collection of ST-7827 genomes (Figure S2). Previous work has shown that ST-7827 is made up of multiple clades,^47,48^ possibly due to recombination-mediated ST reassignments, suggesting that Ethiopian ST-7827 is not related by transmission to global ST-7827 isolates.

The global diversity analysis was constrained by the sparse sampling of *N. gonorrhoeae* genomes from sub-Saharan Africa. Expanded sampling in the region may reveal additional introductions and transmission patterns not detected in this study.

In conclusion, this study reports the genetic diversity, AMR determinants, and phylogenetic relationships of *N. gonorrhoeae* isolates circulating in Bahir Dar. Our findings underscore the need for continuous genomic monitoring and enhanced antimicrobial stewardship to mitigate the threat posed by multidrug-resistant *N. gonorrhoeae*. Expanding the geographic and temporal scope of WGS-based surveillance will further elucidate transmission and resistance trends within Ethiopia and contribute to global efforts to control gonococcal infections.

## Supplementary Material

dlag126_Supplementary_Data

## Data Availability

The genome sequences have been deposited in the PubMLST.org database (http://pubmlst.org/neisseria/), identification numbers and PubMLST isolate names are available in Table S1. The code for the phylogenetic analysis in the global context is available at: https://github.com/gradlab/ethiopia_gc.

## Supplementary data

Figures S1 to S3 and Tables S1 to S7 are available as Supplementary data at *JAC-AMR* Online.
