# Supplementary material for: Phenotypic and genotypic characterization of Neisseria gonorrhoeae isolates from Ethiopia, 2021 to 2023
Source: JAC Antimicrob Resist. 2026 Jul 23;8(4):dlag126. doi: 10.1093/jacamr/dlag126 (PMC13392472; doi:10.1093/jacamr/dlag126)
Supplement: dlag126_Supplementary_Data [file dlag126_supplementary_data.zip › SUPPLEMENTARY,_Derebe_et_al.docx]

**SUPPLEMENTARY DATA**

**Phenotypic and genotypic characterization of *Neisseria gonorrhoeae* isolates from Ethiopia, 2021 to 2023**

Mulatu Melese Derebe^1,2^, QinQin Yu^4^, Abaineh Munshea^1^, Gizachew Yismaw Wubetu^2^, Surafel Fentaw^3^, Tesfa Addis^3^, Rebecca M McSweeney^4^, Nadia Debech^6^, Vegard Eldholm^6^, Tatiana Ponton Tomaselli^6^, Afework Kassu^8^, Adane Mihret^8^, Yemane Berhane^7^, Anne CC Lee,^5*^ Yonatan H Grad^4*^, Bente Børud^6*^†

^1^Health Biotechnology Division, Institute of Biotechnology, Bahir Dar University, Bahir Dar, Ethiopia

^2^Amhara Public Health Institute, Bahir Dar, Ethiopia

^3^Ethiopian Public Health Institute, Addis Ababa, Ethiopia

^4^Department of Immunology and Infectious Diseases, Harvard T.H. Chan School of Public Health, Boston, USA

^5^Global Alliance for Infant and Maternal Health Research, Warren Alpert Medical School, Department of Pediatrics, Brown University, Providence, RI, USA

^6^Department of Bacteriology, Norwegian Institute of Public Health, Oslo, Norway

^7^Addis Continental Institute of Public Health, Addis Ababa, Ethiopia

^8^Armauer Hansen Research Institute, Addis Ababa, Ethiopia

* Joint senior authors

†Corresponding author: Bente Børud, Norwegian Institute of Public Health, Oslo, Norway, Email: [Bente.Borud@fhi.no](mailto:Bente.Borud@fhi.no), Phone: +47 41440530

**Short running title:** Gonococcal phylogeny and AMR determinants in Ethiopia.

**Keywords:** *Neisseria gonorrhoeae*, antimicrobial resistance, bacterial genomics.


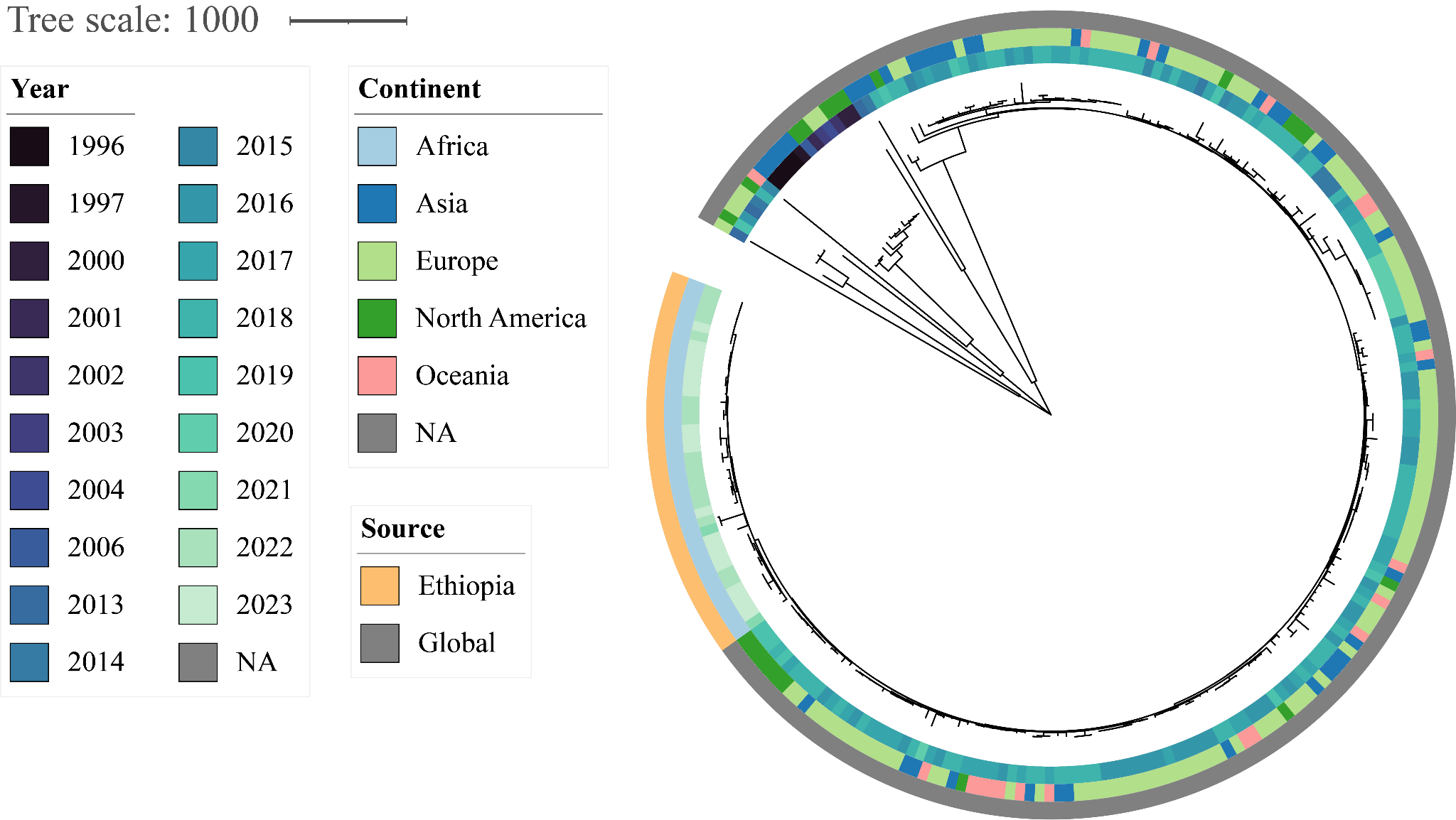


**Figure S1.** Recombination-corrected phylogeny of *N. gonorrhoeae* ST-1587 isolates from Bahir Dar, Ethiopia, in comparison with all publicly available global ST-1587 isolates. The tree scale bar indicates the number of recombination-corrected mutations.


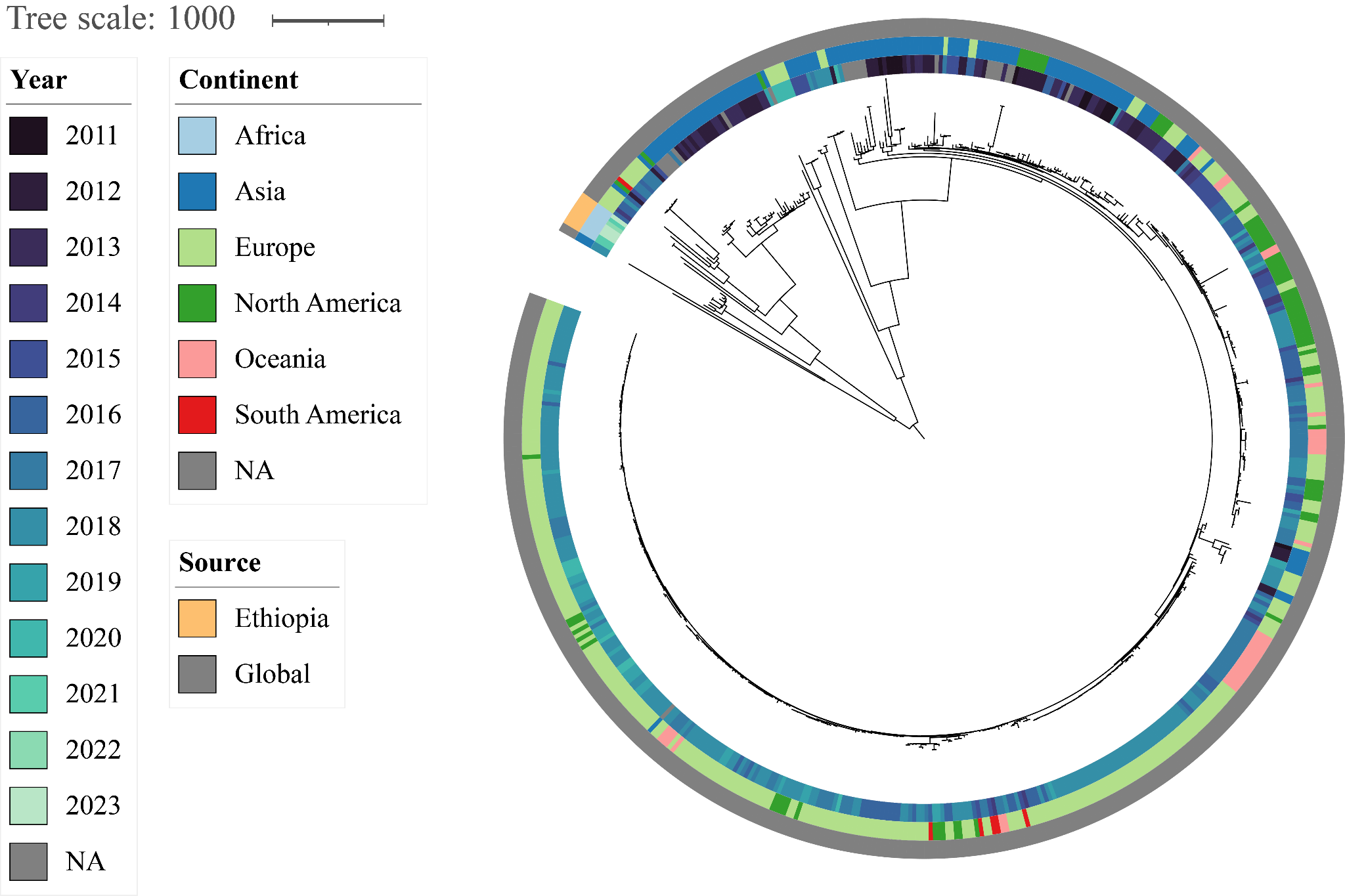


**Figure S2.** Recombination-corrected phylogeny of *N. gonorrhoeae* sequence type ST-7827 isolates from Bahir Dar, Ethiopia, in comparison with all publicly available global ST-7827 isolates. The tree scale bar indicates the number of recombination-corrected mutations**.**


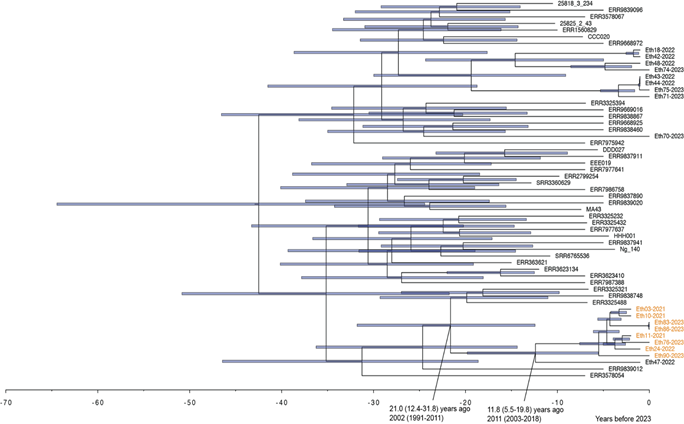


**Figure S3.** Dated phylogeny of *N. gonorrhoeae* ST-7827 isolates from Bahir Dar, Ethiopia, compared with the phylogenetically closest global representatives and closest Ethiopian isolates.

*The isolates included in this phylogeny are indicated by the gray shading in Figure 3. The blue bars indicate the 95% highest posterior density (HPD) of the node times. The ST-7827 Bahir Dar isolates are highlighted in orange. The estimated divergence time of the ST-7827 Bahir Dar isolates from the closest non-ST-7827 Ethiopian isolate is 2012 (11.8 years before 2023), with a 95% HPD density between 2004 and 2018 (5.5-19.8 years before 2023). The estimated divergence time of the ST-7827 Bahir Dar isolates from the closest representative global isolates is 2002 (21 years before 2023), with 95% HPD between 1991 and 2011.

**Table S2:** References and number of isolates for publicly available genomes that met quality control thresholds for inclusion in the study.

| **Reference** | **Number of isolates** | **Reference** | **Number of isolates** |
| --- | --- | --- | --- |
| Alfsnes et al., 2020  ^1^ | 840 | Lan et al., 2020  ^2^ | 228 |
| Bristow et al., 2023  ^3^ | 444 | Lee et al., 2018  ^4^ | 392 |
| Buckley et al., 2018  ^5^ | 43 | Liao et al., 2023  ^6^ | 10 |
| Cehovin et al., 2018  ^7^ | 73 | Liu et al., 2022  ^8^ | 13 |
| Day et al., 2022  ^9^ | 1 | Mitchev et al., 2021  ^10^ | 61 |
| De Silva et al., 2016  ^11^ | 1400 | Mortimer et al., 2021  ^12^ | 894 |
| Demczuk et al., 2015  ^13^ | 90 | Parmar et al., 2020  ^14^ | 56 |
| Demczuk et al., 2016  ^15^ | 187 | Peng et al., 2019  ^16^ | 431 |
| Didelot et al., 2016  ^17^ | 224 | Pinto et al., 2020  ^18^ | 421 |
| Eyre et al., 2017  ^19^ | 231 | Reimche et al., 2021  ^20^ | 1014 |
| Ezewudo et al., 2015  ^21^ | 51 | Reimche et al., 2023  ^22^ | 1298 |
| Fifer et al., 2018  ^23^ | 46 | Ryan et al., 2018  ^24^ | 34 |
| Gernert et al., 2020  ^25^ | 319 | Salmeron et al., 2021  ^26^ | 15 |
| Gianecini et al., 2023  ^27^ | 11 | Sanchez-Buso et al., 2019  ^28^ | 382 |
| Golparian et al., JAC, 2020  ^29^ | 511 | Sanchez-Buso et al., 2022  ^30^ | 2363 |
| Golparian et al., 2020  ^31^ | 189 | Sangprasert et al., 2024  ^32^ | 2 |
| Gianecini et al., 2019  ^33^ | 156 | Thomas et al., 2019  ^34^ | 311 |
| Golparian et al., 2022  ^35^ | 61 | Town et al., 2021  ^36^ | 1265 |
| Golparian et al., 2023  ^37^ | 1 | Jamoralin et al., 2021  ^38^ | 13 |
| Grad et al., 2014  ^39^ | 1093 | Wang et al., 2023  ^40^ | 616 |
| Hadad et al., 2021  ^41^ | 1109 | Williamson et al., 2019  ^42^ | 2109 |
| Harris et al., 2018  ^43^ | 1047 | Yahara et al., 2018  ^44^ | 252 |
| Kong et al., 2021  ^45^ | 335 | Yahara et al., 2021  ^46^ | 208 |
| De Korne-Elenbaas et al., 2021  ^47^ | 311 | Zhang et al., 2023  ^48^ | 7 |
| Kwong et al., 2017  ^49^ | 94 | Zondag et al., 2023  ^50^ | 321 |
| Total | 21583 | | |

**Table S3:** Patients’ characteristics for *Neisseria gonorrhoeae* infections (n=70) in Bahir Dar, Ethiopia (2021-2023)

| **Variables** | **Mean (SD) or n (%)** |
| --- | --- |
| **Age (yr)** |  |
| Mean | 26.71 |
| Range | 20 |
| SD | 5.88 |
| **Sex** |  |
| Male | 42 (60%) |
| Female | 28 (40%) |
| **Specimen source** |  |
| Urethral | 42 (60%) |
| Endocervical | 28 (40%) |
| **Sexual partner within the last 6 months** |  |
| 1 | 22 (31.4%) |
| 2 | 16 (22.9%) |
| 3 | 9 (12.9%) |
| ≥4 | 23 (32.9%) |
| **Travel history in the last two weeks (local)** |  |
| No travel | 57 (81.4%) |
| One time | 3 (4.3%) |
| ≥2 times | 10 (14.3%) |

**Table S4:** MLST Sequence Types with health facility distribution in *N. gonorrhoeae* isolates collected from Bahir Dar, Ethiopia (2021-2023).

| **MLST**  **STs** | **FGAE** | **Agmas clinic** | **Africa Clinic** | **Adidas Hospital** | **Tabor Clinic** | **Dream Care Hospital** | **B/ Dar HC** | **Han HC** | **GAMBY Hospital** | **Eyasta Med. Center** | **Total**  **%** |
| --- | --- | --- | --- | --- | --- | --- | --- | --- | --- | --- | --- |
| **1587** | 13 | 9 | 2 | - | 2 | 2 | 2 | 4 | 2 | 2 | 38  54.3% |
| **7827** | 4 | 1 | 1 | 1 | - | - | - | 1 | - | - | 8  11.4% |
| **11257** | 2 | - | - | - | 1 | - | - | - | 1 |  | 4  5.7% |
| **8133** | - | 3 | - | - | - | - | - | - | - | - | 3  4.3% |
| **11251** | 1 | 1 | 1 | - | - | - | - | - | - | - | 3  4.3% |
| **13781** | 1 | - | 1 | - | 1 | - | - | - | - | - | 3  4.3% |
| **8775** | 1 | 1 | - | - | - | - | - | - | - | - | 2  2.9% |
| **18035** | 1 | - | - | - | - | - | 1 | - | - | - | 2  2.9% |
| **18060** | 1 | - | - | - | - | - | 1 | - | - | - | 2  2.9% |
| **1918** | - | - | - | 1 | - | - | - | - | - | - | 1  1.4% |
| **8134** | 1 | - | - | - | - | - | - | - | - | - | 1  1.4% |
| **11249** | - | - | - | - | - | 1 | - | - | - | - | 1  1.4% |
| **11365** | 1 | - | - | - | - | - | - | - | - | - | 1  1.4% |
| **13333** | - | 1 | - | - | - | - | - | - | - | - | 1  1.4% |
| **Total**  **%** | 26 37.1% | 16 22.8% | 5  7.1% | 2  2.9% | 4  5.7% | 3  4.3% | 4  5.7% | 5  7.1% | 3  4.3% | 2  2.9% | 70  100% |

**Table S5.** Sequence type frequency with gender distribution from MLST in *N. gonorrhoea* isolates collected from Bahir Dar, Ethiopia (2021-2023).

| **MLST Sequence Type** | **No of isolates** | **Frequency (%)** | **Gender** | |
| --- | --- | --- | --- | --- |
|  |  |  | **Male** | **Female** |
| **1587** | 38 | 54.29 | 21 | 17 |
| **7827** | 8 | 11.43 | 5 | 3 |
| **11257** | 4 | 5.71 | 2 | 2 |
| **8133** | 3 | 4.29 | 3 | 0 |
| **11251** | 3 | 4.29 | 2 | 1 |
| **13781** | 3 | 4.29 | 3 | 0 |
| **18060** | 2 | 2.86 | 0 | 2 |
| **8775** | 2 | 2.86 | 1 | 1 |
| **18035** | 2 | 2.86 | 1 | 1 |
| **1918** | 1 | 1.43 | 1 | 0 |
| **8134** | 1 | 1.43 | 1 | 0 |
| **11365** | 1 | 1.43 | 0 | 1 |
| **11249** | 1 | 1.43 | 1 | 0 |
| **13333** | 1 | 1.43 | 1 | 0 |
| **Total** | 70 | 100 | 42 | 28 |

**Table S6.** NG-STAR**-**Sequence Type frequencies with gender distribution in *N. gonorrhoea* isolates collected from Bahir Dar, Ethiopia (2021-2023)

| **NG-STAR Sequence Type** | **No isolates**  **(n=70)** | **Frequency (%)** | **Gender** | |
| --- | --- | --- | --- | --- |
|  |  |  | **Male** | **Female** |
| 5621 | 13 | 18.57 | 4 | 9 |
| 1203 | 10 | 14.29 | 5 | 5 |
| 5666 | 7 | 10.00 | 5 | 2 |
| 4757 | 4 | 5.71 | 3 | 1 |
| 5627 | 4 | 5.71 | 4 | 0 |
| 1054 | 3 | 4.29 | 3 | 0 |
| 5623 | 3 | 4.29 | 3 | 0 |
| 5635 | 3 | 4.29 | 1 | 2 |
| 5606 | 2 | 2.86 | 0 | 2 |
| 5611 | 2 | 2.86 | 2 | 0 |
| 5616 | 2 | 2.86 | 1 | 1 |
| 5617 | 2 | 2.86 | 1 | 1 |
| 5628 | 2 | 2.86 | 2 | 0 |
| 5678 | 2 | 2.86 | 1 | 1 |
| 6038 | 2 | 2.86 | 1 | 1 |
| 5620 | 1 | 1.43 | 0 | 1 |
| 5622 | 1 | 1.43 | 1 | 0 |
| 5626 | 1 | 1.43 | 1 | 0 |
| 5633 | 1 | 1.43 | 1 | 0 |
| 5668 | 1 | 1.43 | 1 | 0 |
| 5670 | 1 | 1.43 | 0 | 1 |
| 5676 | 1 | 1.43 | 1 | 0 |
| 5679 | 1 | 1.43 | 1 | 0 |
| 6043 | 1 | 1.43 | 0 | 1 |
| Total | 70 | 100 | 42 | 28 |

**Table S7.** NG-STAR-Sequence Types with facility distribution of *N. gonorrhoeae* isolates collected from Bahir Dar, Ethiopia (2021-2023)

| **NG-STAR**  **STs** | **FGAE** | **Agmas clinic** | **Africa Clinic** | **Adidas Hospital** | **Tabor Clinic** | **Dream Care Hospit** | **B/ Dar HC** | **Han HC** | **GAMBY Hospital** | **Eyasta Medical Center** | **Total (%)** |
| --- | --- | --- | --- | --- | --- | --- | --- | --- | --- | --- | --- |
| 5621 | 7 | 1 | - | - | - | - | 1 | 3 | - | 1 | 13 (18.6%) |
| 1203 | 3 | 3 | - | - | 1 | - | - | 1 | 1 | 1 | 10 (14.3%) |
| 5666 | 2 | 3 | - | 1 | - | 1 | - | - | - | - | 7 (10%) |
| 4757 | 2 | 1 | - | - | - | - | - | 1 | - | - | 4 (5.7%) |
| 5627 | - | 2 | 1 | - | - | - | - | - | 1 | - | 4 (5.7%) |
| 1054 | 1 | - | 1 |  | 1 | - | - | - | - | - | 3 (4.3%) |
| 5623 | - | 3 | - | - | - | - | - | - | - | - | 3 (4.3%) |
| 5635 | 2 | - | - | - | 1 | - | - | - | - | - | 3 (4.3%) |
| 5606 | 1 | - | - | - | - | - | 1 | - | - | - | 2 (2.9%) |
| 5611 | - | 1 | - | - | - | - | 1 | - | - | - | 2 (2.9%) |
| 5616 | 1 | - | 1 | - | - | - | - | - | - | - | 2 (2.9%) |
| 5617 | 1 | 1 | - | - | - | - | - | - | - | - | 2 (2.9%) |
| 5628 | - | - | 1 | - | - | 1 | - | - | - | - | 2 (2.9%) |
| 5678 | 1 | - | - | 1 | - | - | - | - | - | - | 2 (2.9%) |
| 6038 | 1 | - | 1 | - | - | - | - | - | - | - | 2 (2.9%) |
| 5620 | 1 | - | - | - | - | - | - | - | - | - | 1 (1.4%) |
| 5622 | - | 1 | - | - | - | - | - | - | - | - | 1 (1.4%) |
| 5626 | - | - | - | - | - | 1 | - | - | - | - | 1 (1.4%) |
| 5633 | - | - | - | - | - | - | - | - | 1 |  | 1 (1.4%) |
| 5668 | - | - | - | - | - | - | 1 | - | - | - | 1 (1.4%) |
| 5670 | 1 | - | - | - | - | - | - | - | - | - | 1 (1.4%) |
| 5676 | - | - | - | - | 1 | - | - | - | - | - | 1 (1.4%) |
| 5679 | 1 | - | - | - | - | - | - | - | - | - | 1 (1.4%) |
| 6043 | 1 | - | - | - | - | - | - | - | - | - | 1 (1.4%) |
| Total | 26 | 16 | 5 | 2 | 4 | 3 | 4 | 5 | 3 | 2 | 70 (100%) |

**References**

1 Alfsnes, K., Eldholm, V., Olsen, A. O. *et al.* Genomic epidemiology and population structure of Neisseria gonorrhoeae in Norway, 2016-2017. *Microb Genom* 6 (2020). <https://doi.org/10.1099/mgen.0.000359>

2 Lan, P. T., Golparian, D., Ringlander, J. *et al.* Genomic analysis and antimicrobial resistance of Neisseria gonorrhoeae isolates from Vietnam in 2011 and 2015-16. *The Journal of antimicrobial chemotherapy* 75, 1432-1438 (2020). <https://doi.org/10.1093/jac/dkaa040>

3 Bristow, C. C., Mortimer, T. D., Morris, S. *et al.* Whole-Genome Sequencing to Predict Antimicrobial Susceptibility Profiles in Neisseria gonorrhoeae. *The Journal of infectious diseases* 227, 917-925 (2023). <https://doi.org/10.1093/infdis/jiad027>

4 Lee, R. S., Seemann, T., Heffernan, H. *et al.* Genomic epidemiology and antimicrobial resistance of Neisseria gonorrhoeae in New Zealand. *The Journal of antimicrobial chemotherapy* 73, 353-364 (2018). <https://doi.org/10.1093/jac/dkx405>

5 Buckley, C., Forde, B. M., Trembizki, E. *et al.* Use of whole genome sequencing to investigate an increase in Neisseria gonorrhoeae infection among women in urban areas of Australia. *Sci Rep* 8, 1503 (2018). <https://doi.org/10.1038/s41598-018-20015-x>

6 Liao, Y., Xie, Q., Li, X. *et al.* Dissemination of Neisseria gonorrhoeae with decreased susceptibility to extended-spectrum cephalosporins in Southern China, 2021: a genome-wide surveillance from 20 cities. *Ann Clin Microbiol Antimicrob* 22, 39 (2023). <https://doi.org/10.1186/s12941-023-00587-x>

7 Cehovin, A., Harrison, O. B., Lewis, S. B. *et al.* Identification of Novel Neisseria gonorrhoeae Lineages Harboring Resistance Plasmids in Coastal Kenya. *The Journal of infectious diseases* 218, 801-808 (2018). <https://doi.org/10.1093/infdis/jiy240>

8 Liu, H., Tang, K., Pham, C. D. *et al.* Characterization of a Neisseria gonorrhoeae Ciprofloxacin panel for an antimicrobial resistant Isolate Bank. *PloS one* 17, e0264149 (2022). <https://doi.org/10.1371/journal.pone.0264149>

9 Day, M., Pitt, R., Mody, N. *et al.* Detection of 10 cases of ceftriaxone-resistant Neisseria gonorrhoeae in the United Kingdom, December 2021 to June 2022. *Euro Surveill* 27 (2022). <https://doi.org/10.2807/1560-7917.ES.2022.27.46.2200803>

10 Mitchev, N., Singh, R., Allam, M. *et al.* Antimicrobial Resistance Mechanisms, Multilocus Sequence Typing, and NG-STAR Sequence Types of Diverse Neisseria gonorrhoeae Isolates in KwaZulu-Natal, South Africa. *Antimicrobial agents and chemotherapy* 65, e0075921 (2021). <https://doi.org/10.1128/AAC.00759-21>

11 De Silva, D., Peters, J., Cole, K. *et al.* Whole-genome sequencing to determine transmission of Neisseria gonorrhoeae: an observational study. *The Lancet infectious diseases* 16, 1295-1303 (2016). <https://doi.org/10.1016/S1473-3099(16)30157-8>

12 Mortimer, T. D., Pathela, P., Crawley, A. *et al.* The Distribution and Spread of Susceptible and Resistant Neisseria gonorrhoeae Across Demographic Groups in a Major Metropolitan Center. *Clinical infectious diseases : an official publication of the Infectious Diseases Society of America* 73, e3146-e3155 (2021). <https://doi.org/10.1093/cid/ciaa1229>

13 Demczuk, W., Lynch, T., Martin, I. *et al.* Whole-genome phylogenomic heterogeneity of Neisseria gonorrhoeae isolates with decreased cephalosporin susceptibility collected in Canada between 1989 and 2013. *Journal of clinical microbiology* 53, 191-200 (2015). <https://doi.org/10.1128/JCM.02589-14>

14 Parmar, N. R., Singh, R., Martin, I. *et al.* Genomic Analysis Reveals Antibiotic-Susceptible Clones and Emerging Resistance in Neisseria gonorrhoeae in Saskatchewan, Canada. *Antimicrobial agents and chemotherapy* 64 (2020). <https://doi.org/10.1128/AAC.02514-19>

15 Demczuk, W., Martin, I., Peterson, S. *et al.* Genomic Epidemiology and Molecular Resistance Mechanisms of Azithromycin-Resistant Neisseria gonorrhoeae in Canada from 1997 to 2014. *Journal of clinical microbiology* 54, 1304-1313 (2016). <https://doi.org/10.1128/JCM.03195-15>

16 Peng, J. P., Yin, Y. P., Chen, S. C. *et al.* A Whole-genome Sequencing Analysis of Neisseria gonorrhoeae Isolates in China: An Observational Study. *EClinicalMedicine* 7, 47-54 (2019). <https://doi.org/10.1016/j.eclinm.2019.01.010>

17 Didelot, X., Dordel, J., Whittles, L. K. *et al.* Genomic Analysis and Comparison of Two Gonorrhea Outbreaks. *mBio* 7 (2016). <https://doi.org/10.1128/mBio.00525-16>

18 Pinto, M., Borges, V., Isidro, J. *et al.* Neisseria gonorrhoeae clustering to reveal major European whole-genome-sequencing-based genogroups in association with antimicrobial resistance. *Microb Genom* 7 (2021). <https://doi.org/10.1099/mgen.0.000481>

19 Eyre, D. W., De Silva, D., Cole, K. *et al.* WGS to predict antibiotic MICs for Neisseria gonorrhoeae. *The Journal of antimicrobial chemotherapy* 72, 1937-1947 (2017). <https://doi.org/10.1093/jac/dkx067>

20 Reimche, J. L., Chivukula, V. L., Schmerer, M. W. *et al.* Genomic Analysis of the Predominant Strains and Antimicrobial Resistance Determinants Within 1479 Neisseria gonorrhoeae Isolates From the US Gonococcal Isolate Surveillance Project in 2018. *Sex Transm Dis* 48, S78-S87 (2021). <https://doi.org/10.1097/OLQ.0000000000001471>

21 Ezewudo, M. N., Joseph, S. J., Castillo-Ramirez, S. *et al.* Population structure of Neisseria gonorrhoeae based on whole genome data and its relationship with antibiotic resistance. *PeerJ* 3, e806 (2015). <https://doi.org/10.7717/peerj.806>

22 Reimche, J. L., Clemons, A. A., Chivukula, V. L. *et al.* Genomic analysis of 1710 surveillance-based Neisseria gonorrhoeae isolates from the USA in 2019 identifies predominant strain types and chromosomal antimicrobial-resistance determinants. *Microb Genom* 9 (2023). <https://doi.org/10.1099/mgen.0.001006>

23 Fifer, H., Cole, M., Hughes, G. *et al.* Sustained transmission of high-level azithromycin-resistant Neisseria gonorrhoeae in England: an observational study. *The Lancet infectious diseases* 18, 573-581 (2018). <https://doi.org/10.1016/S1473-3099(18)30122-1>

24 Ryan, L., Golparian, D., Fennelly, N. *et al.* Antimicrobial resistance and molecular epidemiology using whole-genome sequencing of Neisseria gonorrhoeae in Ireland, 2014-2016: focus on extended-spectrum cephalosporins and azithromycin. *Eur J Clin Microbiol Infect Dis* 37, 1661-1672 (2018). <https://doi.org/10.1007/s10096-018-3296-5>

25 Gernert, K. M., Seby, S., Schmerer, M. W. *et al.* Azithromycin susceptibility of Neisseria gonorrhoeae in the USA in 2017: a genomic analysis of surveillance data. *Lancet Microbe* 1, e154-e164 (2020). <https://doi.org/10.1016/S2666-5247(20)30059-8>

26 Salmeron, P., Vinado, B., Arando, M. *et al.* Neisseria gonorrhoeae antimicrobial resistance in Spain: a prospective multicentre study. *The Journal of antimicrobial chemotherapy* 76, 1523-1531 (2021). <https://doi.org/10.1093/jac/dkab037>

27 Gianecini, R. A., Poklepovich, T., Golparian, D. *et al.* Sustained Transmission of Neisseria gonorrhoeae Strains with High-Level Azithromycin Resistance (MIC >/= 256 mug/mL) in Argentina, 2018 to 2022. *Microbiol Spectr* 11, e0097023 (2023). <https://doi.org/10.1128/spectrum.00970-23>

28 Sanchez-Buso, L., Golparian, D., Corander, J. *et al.* The impact of antimicrobials on gonococcal evolution. *Nat Microbiol* 4, 1941-1950 (2019). <https://doi.org/10.1038/s41564-019-0501-y>

29 Golparian, D., Bazzo, M. L., Golfetto, L. *et al.* Genomic epidemiology of Neisseria gonorrhoeae elucidating the gonococcal antimicrobial resistance and lineages/sublineages across Brazil, 2015-16. *The Journal of antimicrobial chemotherapy* 75, 3163-3172 (2020). <https://doi.org/10.1093/jac/dkaa318>

30 Sanchez-Buso, L., Cole, M. J., Spiteri, G. *et al.* Europe-wide expansion and eradication of multidrug-resistant Neisseria gonorrhoeae lineages: a genomic surveillance study. *Lancet Microbe* 3, e452-e463 (2022). <https://doi.org/10.1016/S2666-5247(22)00044-1>

31 Golparian, D., Harris, S. R., Sanchez-Buso, L. *et al.* Genomic evolution of Neisseria gonorrhoeae since the preantibiotic era (1928-2013): antimicrobial use/misuse selects for resistance and drives evolution. *BMC Genomics* 21, 116 (2020). <https://doi.org/10.1186/s12864-020-6511-6>

32 Sangprasert, P., Golparian, D., Paopang, P. *et al.* Complete reference genomes of two ceftriaxone-resistant Neisseria gonorrhoeae strains identified in routine surveillance in Bangkok, Thailand, using Nanopore Q20+ chemistry, VolTRAX V2b, and Illumina sequencing. *Microbiol Resour Announc* 13, e0123123 (2024). <https://doi.org/10.1128/mra.01231-23>

33 Gianecini, R. A., Golparian, D., Zittermann, S. *et al.* Genome-based epidemiology and antimicrobial resistance determinants of Neisseria gonorrhoeae isolates with decreased susceptibility and resistance to extended-spectrum cephalosporins in Argentina in 2011-16. *The Journal of antimicrobial chemotherapy* 74, 1551-1559 (2019). <https://doi.org/10.1093/jac/dkz054>

34 Thomas, J. C., Seby, S., Abrams, A. J. *et al.* Evidence of Recent Genomic Evolution in Gonococcal Strains With Decreased Susceptibility to Cephalosporins or Azithromycin in the United States, 2014-2016. *The Journal of infectious diseases* 220, 294-305 (2019). <https://doi.org/10.1093/infdis/jiz079>

35 Golparian, D., Kittiyaowamarn, R., Paopang, P. *et al.* Genomic surveillance and antimicrobial resistance in Neisseria gonorrhoeae isolates in Bangkok, Thailand in 2018. *The Journal of antimicrobial chemotherapy* 77, 2171-2182 (2022). <https://doi.org/10.1093/jac/dkac158>

36 Town, K., Learner, E. R., Chivukula, V. L. *et al.* Exploring and Comparing the Structure of Sexual Networks Affected by Neisseria gonorrhoeae Using Sexual Partner Services Investigation and Genomic Data. *Sex Transm Dis* 48, S131-S136 (2021). <https://doi.org/10.1097/OLQ.0000000000001520>

37 Golparian, D., Vestberg, N., Sodersten, W. *et al.* Multidrug-resistant Neisseria gonorrhoeae isolate SE690: mosaic penA-60.001 gene causing ceftriaxone resistance internationally has spread to the more antimicrobial-susceptible genomic lineage, Sweden, September 2022. *Euro Surveill* 28 (2023). <https://doi.org/10.2807/1560-7917.ES.2023.28.10.2300125>

38 Jamoralin, M. C., Argimón, S., Lagrada, M. L. *et al.* Genomic surveillance of

in the Philippines, 2013-2014. *West Pac Surveill Re* 12 (2021). <https://doi.org/10.5365/wpsar.2020.11.1.005>

39 Grad, Y. H., Kirkcaldy, R. D., Trees, D. *et al.* Genomic epidemiology of Neisseria gonorrhoeae with reduced susceptibility to cefixime in the USA: a retrospective observational study. *The Lancet infectious diseases* 14, 220-226 (2014). <https://doi.org/10.1016/S1473-3099(13)70693-5>

40 Wang, D., Li, Y., Zhang, C. *et al.* Genomic epidemiology of Neisseria gonorrhoeae in Shenzhen, China, during 2019-2020: increased spread of ceftriaxone-resistant isolates brings insights for strengthening public health responses. *Microbiol Spectr* 11, e0172823 (2023). <https://doi.org/10.1128/spectrum.01728-23>

41 Hadad, R., Golparian, D., Velicko, I. *et al.* First National Genomic Epidemiological Study of Neisseria gonorrhoeae Strains Spreading Across Sweden in 2016. *Front Microbiol* 12, 820998 (2021). <https://doi.org/10.3389/fmicb.2021.820998>

42 Williamson, D. A., Chow, E. P. F., Gorrie, C. L. *et al.* Bridging of Neisseria gonorrhoeae lineages across sexual networks in the HIV pre-exposure prophylaxis era. *Nat Commun* 10, 3988 (2019). <https://doi.org/10.1038/s41467-019-12053-4>

43 Harris, S. R., Cole, M. J., Spiteri, G. *et al.* Public health surveillance of multidrug-resistant clones of Neisseria gonorrhoeae in Europe: a genomic survey. *The Lancet infectious diseases* 18, 758-768 (2018). <https://doi.org/10.1016/S1473-3099(18)30225-1>

44 Yahara, K., Nakayama, S. I., Shimuta, K. *et al.* Genomic surveillance of Neisseria gonorrhoeae to investigate the distribution and evolution of antimicrobial-resistance determinants and lineages. *Microb Genom* 4 (2018). <https://doi.org/10.1099/mgen.0.000205>

45 Kong, L. Y., Wilson, J. D., Moura, I. B. *et al.* Utility of Whole Genome Sequencing in Assessing and Enhancing Partner Notification of Neisseria gonorrhoeae Infection. *Sex Transm Dis* 48, 773-780 (2021). <https://doi.org/10.1097/OLQ.0000000000001419>

46 Yahara, K., Ma, K. C., Mortimer, T. D. *et al.* Emergence and evolution of antimicrobial resistance genes and mutations in Neisseria gonorrhoeae. *Genome Med* 13, 51 (2021). <https://doi.org/10.1186/s13073-021-00860-8>

47 de Korne-Elenbaas, J., Bruisten, S. M., de Vries, H. J. C.,Van Dam, A. P. Emergence of a Neisseria gonorrhoeae clone with reduced cephalosporin susceptibility between 2014 and 2019 in Amsterdam, The Netherlands, revealed by genomic population analysis. *The Journal of antimicrobial chemotherapy* 76, 1759-1768 (2021). <https://doi.org/10.1093/jac/dkab082>

48 Zhang, L., Hu, L., Li, Y. *et al.* Identification of high-level ceftriaxone-resistant Neisseria gonorrhoeae isolates with diverse penA alleles in Zhejiang, China. *J Glob Antimicrob Resist* 35, 51-55 (2023). <https://doi.org/10.1016/j.jgar.2023.08.007>

49 Kwong, J. C., Chow, E. P. F., Stevens, K. *et al.* Whole-genome sequencing reveals transmission of gonococcal antibiotic resistance among men who have sex with men: an observational study. *Sex Transm Infect* 94, 151-157 (2018). <https://doi.org/10.1136/sextrans-2017-053287>

50 Zondag, H. C. A., de Korne-Elenbaas, J., Bruisten, S. M., de Vries, H. J. C.,van Dam, A. P. Increased clonality among Neisseria gonorrhoeae isolates during the COVID-19 pandemic in Amsterdam, the Netherlands. *Microb Genom* 9 (2023). <https://doi.org/10.1099/mgen.0.000975>
